# Causal relationship between Alzheimer’s disease and prostate cancer: a bidirectional Mendelian randomization analysis

**DOI:** 10.3389/fendo.2024.1354528

**Published:** 2024-03-13

**Authors:** Rongkang Li, Lei Peng, Dashi Deng, Guangzhi Li, Song Wu

**Affiliations:** ^1^ Institute of Urology, Lanzhou University Second Hospital, Lanzhou University, Lanzhou, China; ^2^ Institute of Urology, The Affiliated Luohu Hospital of Shenzhen University, Shenzhen University, Shenzhen, China; ^3^ Institute of Urology, South China Hospital, Health Science Center, Shenzhen University, Shenzhen, China

**Keywords:** Mendelian randomization, Alzheimer’s disease, prostate cancer, GWAS, causal analysis

## Abstract

**Background:**

Previous observational researchers have found an inverse bidirectional link between Alzheimer’s disease (AD) and prostate cancer (PCa); yet, the causative nature of this link remains unclear. To investigate the causal interactions between AD and PCa, a bidirectional Mendelian randomization (MR) analysis was conducted.

**Methods:**

This study comprised two Genome-Wide Association Study (GWAS) summary statistics for AD (17,008 cases and 37,154 controls) and PCa (79,148 cases and 61,106 controls) in individuals of European ancestry. The inverse-variance weighted (IVW) method was employed as the primary approach, while MR-Egger, weighted median, weighted mode, and simple mode served as supplementary methods for estimating the causal effect. To assess pleiotropy, the MR-PRESSO global test and MR-Egger regression were used. Cochran’s Q test was adopted to check heterogeneity, MR Steiger test and the leave-one-out analysis was performed to confirm the robustness and reliability of the results.

**Results:**

The causal association genetically inferred of AD on PCa was found using IVW (OR = 0.974, 95% CI = 0.958-0.991, p = 0.003) in forward MR analysis and the causal association genetically inferred of PCa on AD was not found using IVW (OR = 1.000, 95% CI: 0.954-1.049, P = 0.988) in reverse MR analysis. The sensitivity analysis showed that no pleiotropy and heterogeneity was observed. The leave-one-out analysis showed that the findings were not inordinately affected by any instrumental variables.

**Conclusion:**

The results of this study demonstrated an absence of bidirectional causality between AD and PCa among the European population, suggested that a genetically predicted possibility of decreased PCa risk in AD patients, and no significant genetically predicted causal effect of PCa on AD.

## 1 Introduction

Prostate cancer (PCa) represents a prevalent malignancy in elderly males, especially in Western countries, and ranks as the second most frequent cause of cancer-related mortality in the male population (1). Despite advancements in the therapeutic approaches for PCa, there is considerable geographic variability in its occurrence, with all regions experiencing a consistent annual surge in cases (2). Variations in the disease’s progression are attributed to the pathological diversity and the heterogeneity present within the cancerous cells (3). At the moment of PCa diagnosis, nearly 90% of affected individuals exhibit locoregional advancement of the tumor, which often disqualifies them from undergoing surgical interventions (4). Established risk determinants include age, genetic predisposition, and racial or ethnic origins, while the significance of other potential causative factors is still a subject of debate (5). Considering the significant impact of PCa on a global scale, it is imperative to investigate both protective and risk elements of PCa and to implement prompt interventions, aiming to enhance the prognosis for those diagnosed with the disease.

Alzheimer’s disease (AD) stands as the predominant neurodegenerative condition in the aging population, clinically manifesting through memory-related cognitive decline and pathologically distinguished by the presence of extracellular plaques rich in β-amyloid (Aβ) and intracellular neurofibrillary tangles composed of tau protein. The principal risk factor for AD is advanced age (6–8). Recent research has unveiled a reciprocal negative correlation between Alzheimer’s disease and cancer incidence. Studies have indicated that individuals with AD have their cancer risk halved, while a 35% decrease in the likelihood of AD has been noted among individuals who have had or recovered from cancer (9, 10). Prior observational research has suggested that those afflicted with AD may have a diminished probability of developing prostate cancer (11). And, Sherzai et al. observed that patients with PCa have a lower probability of developing AD (12). Such findings highlight a potential association between AD and PCa.

The majority of research examining the link between AD and PCa has utilized cross-sectional or retrospective designs, with a scarcity of prospective studies conducted. Observational studies have not been able to thoroughly investigate the causal relationship between AD and PCa. These studies often face challenges such as limited participant numbers and the absence of randomization. Mendelian randomization (MR) employs genetic variations, usually in the form of single-nucleotide polymorphisms (SNPs), as tools for evaluating the causal influence of an exposure on an outcome (13–15). The MR approach mirrors the principles of a randomized controlled trial, is structured to circumvent biases stemming from confounders that are not accounted for and to prevent biases due to reverse causality (15, 16). And genetic data for this method are frequently sourced from extensive genome-wide association studies (GWAS). As a result, MR offers a time-efficient and economical strategy for discovering potential causal links (17). In this study, a bidirectional two-sample MR approach was utilized to explore the causal relationships between AD and PCa within European demographic groups.

## 2 Methods

### 2.1 Study design and ethics statement

**Figure 1:**
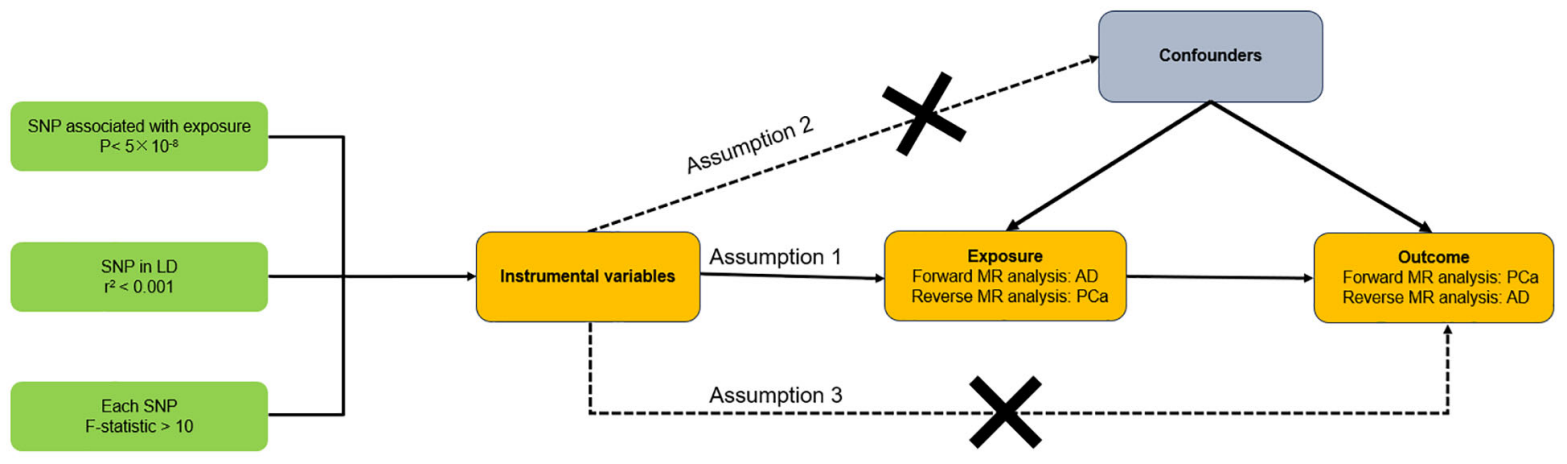
Overall design of this bidirectional Mendelian randomization analysis. AD, Alzheimer’s disease; PCa, Prostate cancer.

**Figure 1** illustrated the schematic of our study design along with the three critical assumptions inherent in the MR study. The first assumption asserts that the instrumental variables (IVs) have a robust association with exposure (AD or PCa). The second assumption contends that the IVs are not affected by confounding variables. The third assumption holds that the IVs exert an effect on the likelihood of exposure exclusively through their interaction with outcome, without any indirect routes (18). Since our data were derived from previously conducted studies or databases available to the public, there was no requirement to obtain additional ethical approval from an ethics committee. In our research, we consistently followed the STROBE-MR guidelines (19, 20), with **Supplementary Material** presenting a checklist that illustrates our adherence to these protocols.

### 2.2 Data source

Our research utilized subjects of European descent from the Integrative Epidemiology Unit (IEU) GWAS database (https://gwas.mrcieu.ac.uk/). All data were extracted from the IEU GWAS database. We identified genetic variants associated with AD by analyzing AD GWAS data (ebi-a-GCST002245) comprising a cohort of 54,162 individuals (21). To avoid overlap between the populations assessed for exposure and outcome, we obtained summary data for prostate cancer from the largest GWAS meta-analysis conducted by the PRACTICAL consortium, which included 79,148 cases and 61,106 controls of European descent (22). The average age of participants with prostate cancer in the PRACTICAL study was 66 years. The distribution of disease stages among these patients was as follows: low aggression, characterized by T0 or T1, Gleason Score ≤6, and PSA <10, accounted for 12.1%; intermediate aggression, defined by T2 or Gleason Score = 7 or PSA between 10 and 20, comprised 37.9%; high aggression, indicated by T3, T4, N1, M1, Gleason Score ≥8, or PSA >20, made up 26.8%; and advanced stages, which included Gleason Score 8+, metastatic disease, PSA >100, or prostate cancer as cause of death according to the previous aggressiveness definition from iCOGS and accessible phenotype data, constituted 20.1% (22). **Table 1** provides comprehensive details about the two sets of GWAS summary data.

**Table 1:** Characteristics of Alzheimer’s disease and prostate cancer GWAS cohorts.

Exposure/Outcome	IEU GWAS id	Cases	Controls	Sample size	Number of SNPs	First Author	Population	PMID
Alzheimer’s disease	ebi-a-GCST002245	17,008	37,154	54,162	7,022,150	Lambert JC	European	24162737
Prostate cancer	ieu-b-85	79,148	61,106	140,254	20,346,368	Schumacher	European	29892016

### 2.3 Selection of instrumental variables

Utilizing the aforementioned GWAS summary data, a rigorous method was adopted to select appropriate SNPs as instrumental variables (IVs). The selection process began with the identification of SNPs that were strongly associated with the exposure, demonstrated by a genome-wide significant P-value threshold of less than 5×10^-8^. To prevent skewed results due to linkage disequilibrium (LD), we applied a clumping procedure with a stringent r^2^ threshold of 0.001 and a 10,000 kb window size. For further refinement, the Phenoscanner database (http://www.phenoscanner.medschl.cam.ac.uk/) was employed to filter out genetic variants linked to potential confounders. In cases where the identified SNPs were absent in the outcome GWAS dataset, we sought substitute proxy SNPs that exhibited a high LD (r^2^ > 0.8) with the initial SNPs. Furthermore, to eliminate confounding from palindromic and ambiguous SNPs with mismatching alleles, we harmonized the exposure and outcome data to ensure consistency in the effect alleles between them. In addition, to address the concern of bias due to weak IVs, we assessed the strength of the IVs by calculating the F-statistic, with an F-statistic significantly greater than 10 indicating a reduced risk of weak instrument bias (23, 24).

### 2.4 Statistical analysis

In this study, various techniques were employed to examine and measure the causal relationships and effects between the exposure and the outcome. These techniques encompassed the inverse-variance weighted (IVW) (25), MR-Egger (26), weighted median (27), simple mode (28) and weighted mode (29). The IVW approach, commonly used in MR analyses, has both random-effects and fixed-effects models. It acts as a meta-analytic tool that aggregates the individual Wald ratio estimates from each IV to provide a consolidated estimate of the effect of exposure on the outcome. The IVW method’s estimates are most credible in the absence of heterogeneity and pleiotropy (30). A random-effects model was implemented when substantial heterogeneity was detected among IVs (p < 0.05); otherwise, a fixed-effect model was chosen. The MR-Egger regression is designed to yield reliable estimates even in the presence of pleiotropy among IVs (31). The weighted median technique is capable of producing causal effect estimates provided that less than half of the IVs breach essential MR preconditions (27). The weighted mode method is suitable for MR causal analysis when the majority of the IVs are valid (29). The simple mode approach offers a less robust alternative to IVW. All these analyses were performed and graphically represented using R version 4.3.1, leveraging the “MRPRESSO” and “TwoSampleMR” R packages. A p-value of less than 0.05 was considered statistically significant.

### 2.5 Sensitivity analysis

We utilized the MR-PRESSO global test and the MR-Egger regression to evaluate pleiotropy in the instrumental variables, with pleiotropy indicated by a p-value of less than 0.05 (26, 32). We assessed heterogeneity through Cochran’s Q statistic, deeming it significant at p < 0.05. The MR Steiger test was used to verify the directionality that exposure causes the outcome (33). Additionally, the “leave-one-out” sensitivity analysis was performed to ascertain whether any single SNP might exert undue influence on the aggregate causal inference.

## 3 Results

### 3.1 Selection of instrumental variables

In accordance with rigorous criteria for selecting instrumental SNPs, we chose suitable SNPs as IVs that conformed to three essential assumptions. We pinpointed 13 SNPs with a high correlation to AD, and 89 SNPs with a high correlation to PCa. These SNPs acted as IVs for exposure (AD or PCa), and each SNP worked out as F-statistic greater than 10, suggesting a low chance of weak IV bias. Detailed information of included SNPs was showed in **Supplementary Tables 1**, **2**.

### 3.2 Causal effects of Alzheimer’s disease on prostate cancer

**Figure 2:**
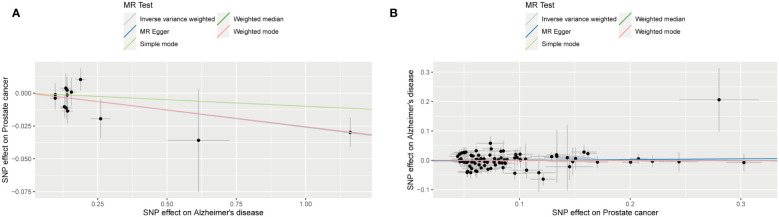
Scatter plot for the causality of Alzheimer’s disease on prostate cancer risk in forward MR analysis **(A)** and Scatter plot for the causality of prostate cancer on Alzheimer’s disease risk in reverse MR analysis **(B)**. The regression slopes of the lines represent the magnitude of the causal effect.

The outcomes of forward MR analysis of the causality of AD on PCa were shown in **Table 2**. IVW revealed a statistically significant negative causal impact of AD on PCa (OR = 0.974, 95% CI = 0.958-0.991, p = 0.003). Simultaneously, a relationship following the same trend was discerned through MR Egger method (OR = 0.974, 95% CI =0.953-0.996, p = 0.039), the weighted median method (OR = 0.975, 95% CI = 0.957-0.993, p = 0.007) and Weighted mode method (OR = 0.975, 95% CI = 0.957-0.993, p = 0.020). These results were graphically represented in both the forest plot (**Supplementary Figure 1**) and the scatter plot (**Figure 2**). The forest plot visually displayed the effect estimates alongside their confidence intervals (CI) for each SNP, whereas the scatter diagram graphically portrayed the association between the exposure (AD) and the outcome (PCa) utilizing the IVs. Given the simple mode approach offers a less robust alternative to IVW, the outcomes from the MR analysis provide support for a causal association between AD and PCa.

**Table 2:** Forward MR analysis of the causality of Alzheimer’s disease on prostate cancer.

Exposure	Outcome	MR method	Number of SNPs	β	SE	OR (95% CI)	P‐value
Alzheimer’s disease	Prostate cancer	MR Egger	13	-0.026	0.011	0.974(0.953-0.996)	0.039
Alzheimer’s disease	Prostate cancer	Weighted median	13	-0.026	0.010	0.975(0.957-0.993)	0.007
Alzheimer’s disease	Prostate cancer	Inverse variance weighted	13	-0.026	0.009	0.974(0.958-0.991)	0.003
Alzheimer’s disease	Prostate cancer	Simple mode	13	-0.010	0.030	0.990(0.933-1.051)	0.748
Alzheimer’s disease	Prostate cancer	Weighted mode	13	-0.026	0.010	0.975(0.957-0.993)	0.020

SNPs, single-nucleotide polymorphisms; SE, standard error; OR, odds ratio; CI, confidence interval.

### 3.3 Causal effects of prostate cancer on Alzheimer’s disease

The outcomes of reverse MR analysis of the causality of PCa on AD were shown in **Table 3**. The findings from the IVW analysis indicated that PCa did not causally influence AD (OR = 1.000, 95% CI: 0.954-1.049, P = 0.988), with the MR-Egger, Weighted Median, Weighted mode and Simple Mode approaches yielding congruent outcomes. The forest plot and the scatter plot were shown in **Supplementary Figure 1** and **Figure 2**. Thus, our results showed that there is no significant causal relationship of PCa on AD.

**Table 3:** Reverse MR analysis of the causality of prostate cancer on Alzheimer’s disease.

Exposure	Outcome	MR method	Number of SNPs	β	SE	OR (95% CI)	P‐value
Prostate cancer	Alzheimer’s disease	MR Egger	89	0.025	0.052	1.025(0.925-1.136)	0.635
Prostate cancer	Alzheimer’s disease	Weighted median	89	-0.006	0.034	0.994(0.929-1.064)	0.869
Prostate cancer	Alzheimer’s disease	Inverse variance weighted	89	0.000	0.024	1.000(0.954-1.049)	0.988
Prostate cancer	Alzheimer’s disease	Simple mode	89	-0.005	0.063	0.995(0.879-1.126)	0.935
Prostate cancer	Alzheimer’s disease	Weighted mode	89	-0.005	0.039	0.995(0.921-1.075)	0.897

SNPs, single-nucleotide polymorphisms; SE, standard error; OR, odds ratio; CI, confidence interval.

### 3.4 Sensitivity analysis

Pleiotropy of IVs was examined through the application of MR-Egger regression and MR-PRESSO global test. The MR-Egger regression inferred that no pleiotropy in IVs (p = 0.994 in forward MR analysis, p = 0.597 in reverse MR analysis, **Table 4**), a conclusion that was further corroborated by the MR-PRESSO global test (p = 0.877 in forward MR analysis, p = 0.132 in reverse MR analysis, **Table 3**). Cochran’s Q test was employed to identify heterogeneity in IVs. The study revealed no heterogeneity in forward MR analysis and reverse MR analysis by the Cochran Q-test (p = 0.729 for MR-Egger, p = 0.799 for IVW in forward MR analysis, p = 0.122 for MR-Egger, p = 0.133 for IVW in reverse MR analysis, **Table 4**) and funnel plots (**Supplementary Figure 2**). The MR Steiger test identified no evidence of reverse causality, and the causal direction was reliable (**Table 4**). A leave-one-out analysis was conducted, sequentially excluding each SNP to assess the impact on the results (**Supplementary Figure 3**). This analysis in forward MR analysis indicated that no single SNP significantly influenced the causal inference, suggesting that the observed overall causal link between AD and PCa was not propelled by any particular SNP, underlining the robustness of the results. These outcomes provide confidence in the validity and robustness of the causal inference derived from the MR analysis.

**Table 4:** Sensitivity analyses of MR.

Exposure	Outcome	Pleiotropy				Heterogeneity				MR Steiger test direction	MR Steiger test P‐value
		MR-PRESSO global outlier test		MR-Egger regression		MR-Egger		Inverse variance weighted (IVW)			
		Rssobs	P‐value	Intercept	P‐value	Q statistic	P‐value	Q statistic	P‐value		
Alzheimer’s disease	Prostate cancer	8.224	0.877	2.585e-05	0.994	7.822	0.729	7.822	0.799	TRUE	8.835e-217
Prostate cancer	Alzheimer’s disease	104.808	0.132	-0.003	0.597	102.536	0.122	102.868	0.133	TRUE	0

Pleiotropy was tesed by MR-PRESSO global outlier test and MR-Egger regression methods. Heterogeneity was tesed by MR-Egger and Inverse variance weighted (IVW) methods. The MR Steiger test was used to detect the reliability of the causal direction.

## 4 Discussion

Previous epidemiological research examining the link between AD and PCa suggests that those diagnosed with AD exhibit a reduced occurrence of PCa, while survivors of PCa show a decreased incidence of AD. Such epidemiological investigations are primarily hypothesis-generating regarding causation and are inevitably constrained by biases and confounding variables. We employed a bidirectional two-sample MR technique to examine the potential causal link between AD and PCa. Analyses using MR methodologies revealed an absence of bidirectional causality between AD and PCa. In particular, our analysis indicated a possibility of decreased PCa risk in AD patients, yet no significant causal effect of PCa on AD was observed. These results offer important perspectives regarding the prospective causal dynamics between the two disorders.

With the rise in the elderly population, there has been a corresponding increase in the prevalence of conditions like cancer and neurodegenerative diseases. Past research focusing mainly on white demographics has found an association between AD and a reduced cancer risk, ranging from 36% to 80%, though this reduction rate differs according to the study design (34–37). Similarly, a retrospective cohort study in Shanghai, China, and a population-based longitudinal study in South Korea have indicated a potential reduced risk for several cancers, included prostate cancer, in patients with AD (11, 38). The results of our study are consistent with these earlier findings.

Several studies have demonstrated an inverse correlation between cancer and AD. An earlier study from Italy indicated that the prevalence of AD did not increase in PC patients (36), while research from Taiwan noted a negligible rise in AD prevalence among PC patients compared to the broader population (39). Individuals who have survived certain cancers appear to have a diminished likelihood of developing AD, rather than other age-related ailments, which suggests that the reduced AD diagnoses are not solely attributable to biases. It has been posited that cancer therapy may be linked to a lower risk of AD (40). Our study showed that no genetic causal association of PCa on AD by MR analysis.

Utilizing discharge information sourced from the National Inpatient Sample (NIS) spanning 1999 to 2008, this subsequent analysis of existing data additionally revealed a reverse correlation between Alzheimer’s disease (AD) and ten types of cancer, where prostate, ovarian, and lung cancers showed the most significant reverse associations (12). Drawing on a cohort study conducted in Northern Italy involving over 1 million residents, it was found that the incidence of cancer in individuals with AD dementia was reduced by half, while the likelihood of developing AD dementia in cancer patients decreased by 35%. The incidence rates for cancers originating from various tissues in individuals with AD dementia were consistently below expected levels; this decrease in risk was statistically significant for cancers arising from epithelial tissues, as well as those from non-epithelial, mesenchymal, blood, or nervous tissues. The number of AD dementia cases among the five common cancer locations (breast, lung, bladder, prostate, colorectal) always fell short of expectations. Nonetheless, the relative risk (RR) showed a significant decline only in individuals with colorectal cancer or with cancers of non-epithelial, mesenchymal, blood, or nervous tissues. The frequency of the five cancer types ((breast, lung, bladder, prostate, colorectal)) in AD dementia patients was below anticipated levels, though the observed prostate cancer cases were nearly the same as expected. Notably, the risk for developing lung and colorectal cancers, along with tumors of non-epithelial, mesenchymal, blood, or nervous tissues, was significantly lower (36). A retrospective cohort study conducted in Shanghai indicates that individuals with AD may have a reduced likelihood of developing various cancers, such as lung, prostate, and testicular cancer. Concurrently, an increased incidence of lymphoma was found to be positively associated with AD (11).

Yuan et al. investigated the bidirectional causality between AD and colorectal cancer (CRC) using a two-sample MR approach. The MR findings indicated a lower likelihood of AD in individuals with CRC, alongside a marginally increased probability of CRC in those diagnosed with AD. However, the reliability of the latter finding is compromised by the effect of overlapping samples (41). Sahba Seddighi and colleagues utilized MR to investigate the causal link between cancer and AD. They found that cancers related to smoking, as predicted genetically, were linked to a 5.2% decrease in the likelihood of AD (OR 0.95, 95% CI 0.92 -0.98, p = 0.0026) for every 1-unit increase in log odds of cancer. Among these, only lung cancer predicted genetically, showing a 9.0% reduction, had a significant association with AD (OR 0.91, 95% CI 0.84-0.99, p = 0.019) per 1-unit increase in log odds of cancer. Cancers not related to smoking, as predicted genetically, were linked to a 1.9% decrease in the odds of AD (OR 0.98, 95% CI 0.97-0.995, p = 0.0091) for each 1-unit increase in log odds of cancer. Within this category, prostate cancer, melanoma, lymphoma, and ovarian cancer, as predicted genetically, showed no significant association with the odds of developing AD. Conversely, leukemia predicted genetically was associated with a 2.4% reduction in AD odds (OR 0.98, 95% CI 0.96- 0.995, p = 0.012) per 1-unit increase in log odds of cancer, and breast cancer was linked to a 5.9% lower likelihood of AD (OR 0.94, 95% CI 0.89-0.99, p = 0.028) for each 1-unit increase in log odds of cancer. Combining genetic predictors for all types of cancer considered in the study, the overall association pointed to a 2.5% reduction in the odds of AD (OR 0.98, 95% CI 0.96-0.99, p = 0.00027) for every 1-unit increase in log odds of cancer (42).

An intriguing theory accounting for the inverse relationship between cancer and AD posits a common etiology that exerts divergent effects on carcinogenesis and neurodegenerative processes (43). Proposed biological pathways include proteins that inhibit tau accumulation and amyloid-β aggregation, as well as those that control cell cycle events (44, 45), common epigenetic alterations (46), and aging-associated disruptions in cell metabolism (47). This hypothesized common etiology offers a significant chance to enhance our understanding of the underlying mechanisms of both carcinogenesis and neurodegenerative conditions (43, 48, 49).

The association between cancer and AD seems to related to disruptions in the regulation of the cell cycle (50). Proteins such as P53, Wnt, and Pin1 are involved in managing the cell cycle for both diseases. The Pin1 gene plays a role in cancer and AD by influencing cell cycle control, signal transduction, DNA damage response, as well as the management of tau and β-amyloid (Aβ) precursor proteins (37, 51). In AD patient brain tissues, Pin1 expression is typically diminished, while in several cancer forms, such as prostate cancer, an increased expression of Pin1 is observed (12, 51–54). Research involving animals indicates that the absence of Pin1 hinders the development of oncogenes and inhibits both tumor and cellular proliferation (12, 53). Simultaneously, a reduced expression of Pin1 may contribute to enhanced neurodegeneration in AD (55). Experiments with animals have shown that elevated Pin1 levels in postnatal neurons can counteract neurodegeneration (51, 56, 57). The depletion of Pin1 in animals results in tau and Aβ-related pathological changes that occur in an age-dependent fashion, mirroring those in AD (12). Due to Pin1’s direct involvement with both cancer and AD, it has garnered considerable interest as a potential therapeutic target and warrants further exploration.

This investigation presented multiple strengths. First, this study utilized MR analysis, a method in genetic epidemiology that applies instrumental variables to assess the causal impact of exposures (AD or PCa) on outcomes (PCa or AD). An earlier MR study reported that prostate cancer did not affect AD (42). However, the bidirectional analysis conducted in this research represents the novelty of the study. Second, the principles of MR analysis ensured resistance to confounder-induced biases and forestalled reverse causality in the MR analysis. The use of published GWAS data provided access to a large cohort and extensive information on genetic diversity. Consequently, the outcomes of this study suggested a potential reduction in PCa risk among individuals with AD, while no significant causal relationship was found from PCa towards AD, implying a notable causal effect of increased AD risk leading to a lower PCa risk. The robustness of these results was further validated through sensitivity analysis. Lastly, the selection of study participants from a European demographic reduced the likelihood of biases related to population stratification.

Nonetheless, this study was subject to certain limitations. First, the GWAS summary statistics were restricted to individuals with European ancestry, hence our conclusions may be primarily applicable to European cohorts. Therefore, extending the application of our findings to racially and ethnically different populations should be done with caution. Second, constraints in our data sources precluded the possibility of performing analyses that were stratified or adjusted for other variables. More exhaustive investigations, such as future prospective randomized controlled trials, could provide deeper insights to the inferences made in this study. Third, the GWAS data for PCa are derived exclusively from males. Unfortunately, GWAS data for AD with gender stratification are not currently available. Consequently, the GWAS data for AD used in this study include both males and females. Ideally, using male-only GWAS data for traits related to AD would be preferable. Lastly, the capability of the MR approach was confined to examining causal links; it did not permit the exploration of the underlying mechanisms behind these associations. Elucidating these mechanisms would necessitate more detailed research.

## 5 Conclusion

In summary, we demonstrated an absence of bidirectional causality between AD and PCa among the European population, employing a bidirectional two-sample MR analysis. Our findings suggested that a genetically predicted possibility of decreased PCa risk in AD patients, and no significant genetically predicted causal effect of PCa on AD. Our results provided new evidence for discovering potential relationship between AD and PCa. Further studies focused on mechanisms are necessary to clarify the complex relationship between AD and PCa.

## Data availability statement

The original contributions presented in the study are included in the article/**Supplementary Material**. Further inquiries can be directed to the corresponding authors.

## Author contributions

RL: Conceptualization, Data curation, Formal analysis, Investigation, Software, Writing – original draft. LP: Conceptualization, Data curation, Formal analysis, Investigation, Software, Writing – original draft. DD: Formal analysis, Software, Writing – original draft. GL: Funding acquisition, Methodology, Project administration, Resources, Supervision, Writing – review & editing. SW: Funding acquisition, Methodology, Project administration, Resources, Supervision, Writing – review & editing.
