# Supplementary material for: Causal relationship between Alzheimer’s disease and prostate cancer: a bidirectional Mendelian randomization analysis
Source: Front Endocrinol (Lausanne). 2024 Mar 13;15:1354528. doi: 10.3389/fendo.2024.1354528 (PMC10965771; doi:10.3389/fendo.2024.1354528)
Supplement: Supplementary file 3 [file Image_1.pdf]

Supplementary Material

1 Supplementary Figures

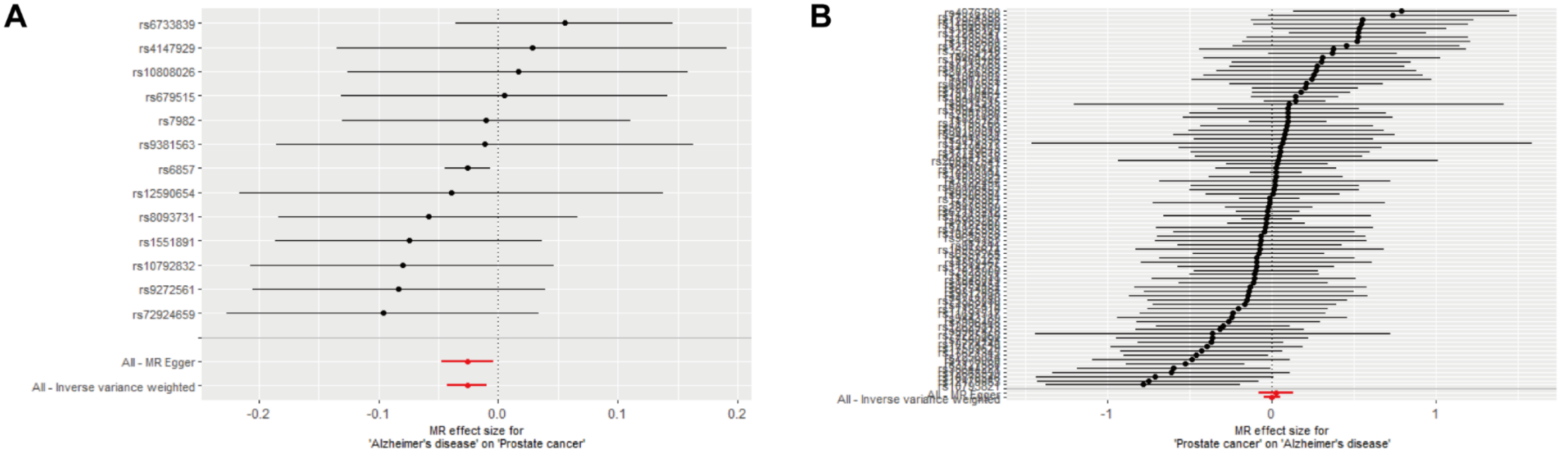

Supplementary Figure 1 Forest plot for the causality of each SNP on Alzheimer's disease risk in forward MR analysis (A) and prostate cancer risk in reverse MR analysis (B).

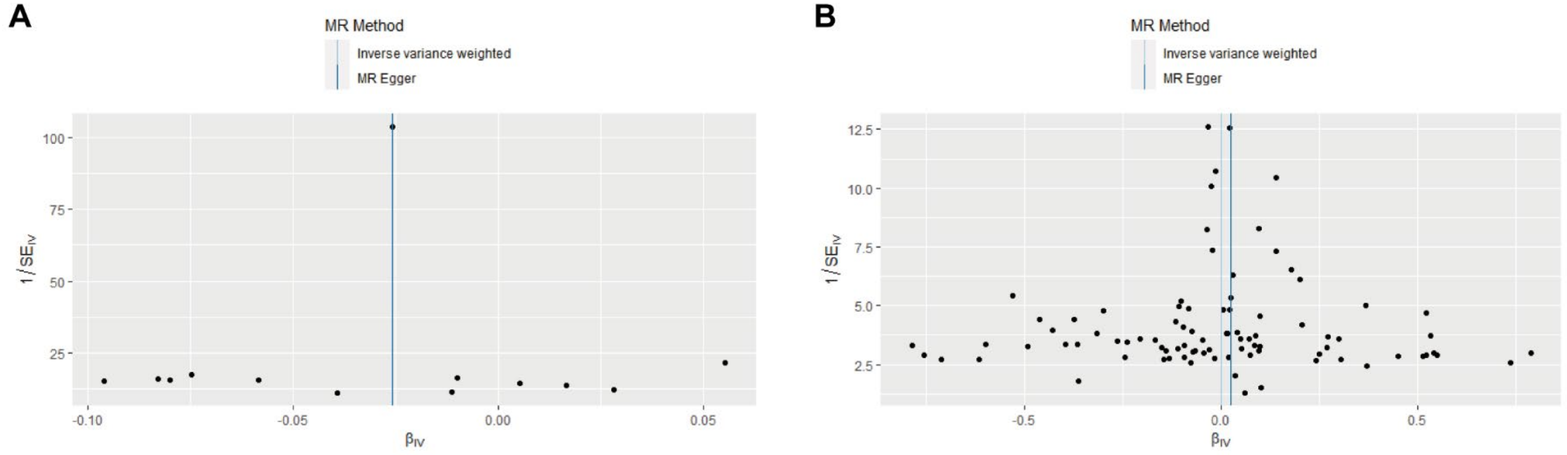

Supplementary Figure 2 Funnel plot for instrumental variables to assess heterogeneity in forward MR analysis (A) and in reverse MR analysis (B).

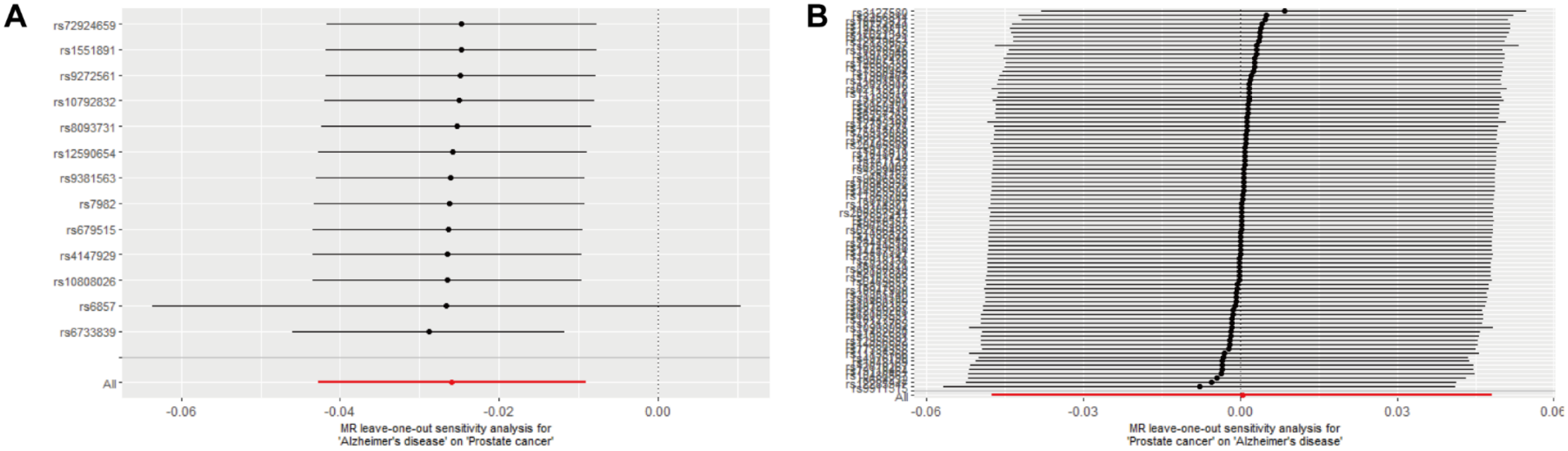

Supplementary Figure 3 Forest plot for leave-one-out analysis of the effect of Alzheimer's disease on prostate cancer in forward MR analysis (A) and prostate cancer on Alzheimer's disease in reverse MR analysis (B).
